# Nursing students’ emotions, educational concerns, and the impact of study careers and professional futures during the COVID-19 pandemic: a cross-sectional study

**DOI:** 10.1186/s12909-024-05231-6

**Published:** 2024-03-13

**Authors:** Miaojing Song, Lin Zhang, Qiqi Ji, Pengjuan Ji, Jiashuang Xu, Yian Chen, Leilei Guo

**Affiliations:** 1 School of Nursing, Jinzhou Medical University, No. 40, Section 3, Songpo Road, Linghe District, Jinzhou City, Liaoning Province PR China; 2 Department of Internal Medicine Nursing, School of Nursing, Wannan Medical College, 22 Wenchang West Road, Higher Education Park, Wuhu City, An Hui Province PR China

**Keywords:** COVID-19, Coronavirus disease 2019, Nursing students, Nursing education

## Abstract

**Background:**

COVID-19 is a challenge to education systems worldwide. The aim of the study was to explore the impact of COVID-19-pandemic-related emotions and COVID-19-related concern for education on the study careers and professional futures of nursing students.

**Methods:**

The study was completed between March and June 2023 using a multi-stage sampling design. A total of 1126 nursing students were recruited to complete the questionnaire. The self-administered questionnaire consisted of basic characteristics of the subjects, the COVID-19-pandemic-related emotions scale, the COVID-19-related concern for education scale, and the impact of the COVID-19 on study careers and professional futures scale (SCPFI-19 S). One-way ANOVA/t-test, correlation coefficient analysis, and hierarchical linear regression analysis were used to explore factors influencing changes in study careers and professional futures, and the relationship between COVID-19-pandemic-related emotions and COVID-19-related concern for education.

**Results:**

Univariate analysis of variance indicated that residence, willingness, and whether to engage in nursing after graduation were related to SCPFI-19 S (P < 0.05). COVID-19-pandemic-related emotions and COVID-19-related concern for education were significantly and positively associated with SCPFI-19 S (r = 0.566, P < 0.01; r = 0.199, P < 0.01). Stratified multiple regression analysis showed that COVID-19-pandemic-related emotions and COVID-19-related concern for education of nursing students were significant predictors of SCPFI-19 S.

**Conclusion:**

Overall, both COVID-19-pandemic-related emotions and COVID-19-related concern for education were significantly correlated with SCPFI-19 S. In future interventions, schools should consider structures and strategies to support students’ mental health and educational trajectories during current and future epidemics or similar crises.

## Background

On January 30, 2020, the World Health Organization declared the new coronavirus (COVID-19) outbreak a public health emergency of international concern [1]. COVID-19 is characterized by a long incubation period, high infectiousness, and rapid outbreaks [2]. It not only poses a huge challenge to our healthcare system but also negatively affects social stability, economic development, and other aspects [3].

In order to prevent the spread of the epidemic on campus, all schools and universities suspended teaching, training, and laboratory activities to avoid direct contact between people and to minimize spread between people in different geographical regions [4]. Inevitably, nursing education has also been affected by the shift from traditional teaching models to online teaching, leading to significant changes in individual lifestyles. This creates a sense of uncertainty and unpredictability that has an impact on people’s psychological states [5]. As the major reserve force of nursing professionals, nursing students are at a learning stage in their fields. Their inadequate understanding of COVID-19 and lack of clinical experience would bring complex emotions [6]. In particular, they have faced a great deal of fear and anxiety due to campus evacuation and the transition to remote learning. Students’ educational processes and learning patterns have also changed, requiring significant resources and increasing their vulnerability [7, 8]. At the same time, undergraduate nursing students were very concerned about their academic progress due to shifts in teaching and assessment patterns [9]. However, some studies have found that negative emotions may lead to decreased motivation and difficulty concentrating, affecting the depth and effectiveness of learning for students [10, 11]. Affect-Cognition Theory suggests that emotions and cognitive processes interact. Emotional states can influence an individual’s cognitive processes and decision-making behavior. During an epidemic, negative emotions (e.g., anxiety, fear) may interfere with the allocation of cognitive resources and information processing, affecting the processing of learning tasks and learning outcomes [12, 13]. Therefore, a deeper understanding of these factors will help us provide targeted support and guidance to help students better cope with negative emotions and succeed in their studies and future careers.

College and university education, i.e., the goal of most young people, is to acquire knowledge or skills that will be decisive for their careers [14]. During the COVID-19 pandemic, online learning has become the norm. Although online learning has been reported to be useful in nursing education, it may be less effective without appropriate infrastructure and systematic learning methods [15]. In addition, the delivery of clinical training is crucial in all nursing education [16]. However, due to the epidemic, clinical practice for nursing trainees was suspended. Many nursing students reported that they would have benefited from clinical placement during their education to further develop their professional competence. Not having the opportunity to practice in the field may negatively impact nursing students’ technical proficiency [17, 18]. Therefore, nursing students have expressed concern about changes to online learning and clinical training, fearing that they will affect the overall quality of their education and their careers after graduation [19]. Self-actualization theory states that individuals’ beliefs and expectations can influence their behavior and level of effort. If individuals worry too much about their education, it may reduce their motivation to aim higher and succeed, which may affect their study career and professional future [20]. Therefore, when examining the relationship between them, we need to gain a deeper understanding of this complexity, identify problems early, and take appropriate measures to lay a solid foundation for the successful development of students’ study careers and professional futures.

**Fig. 1:**
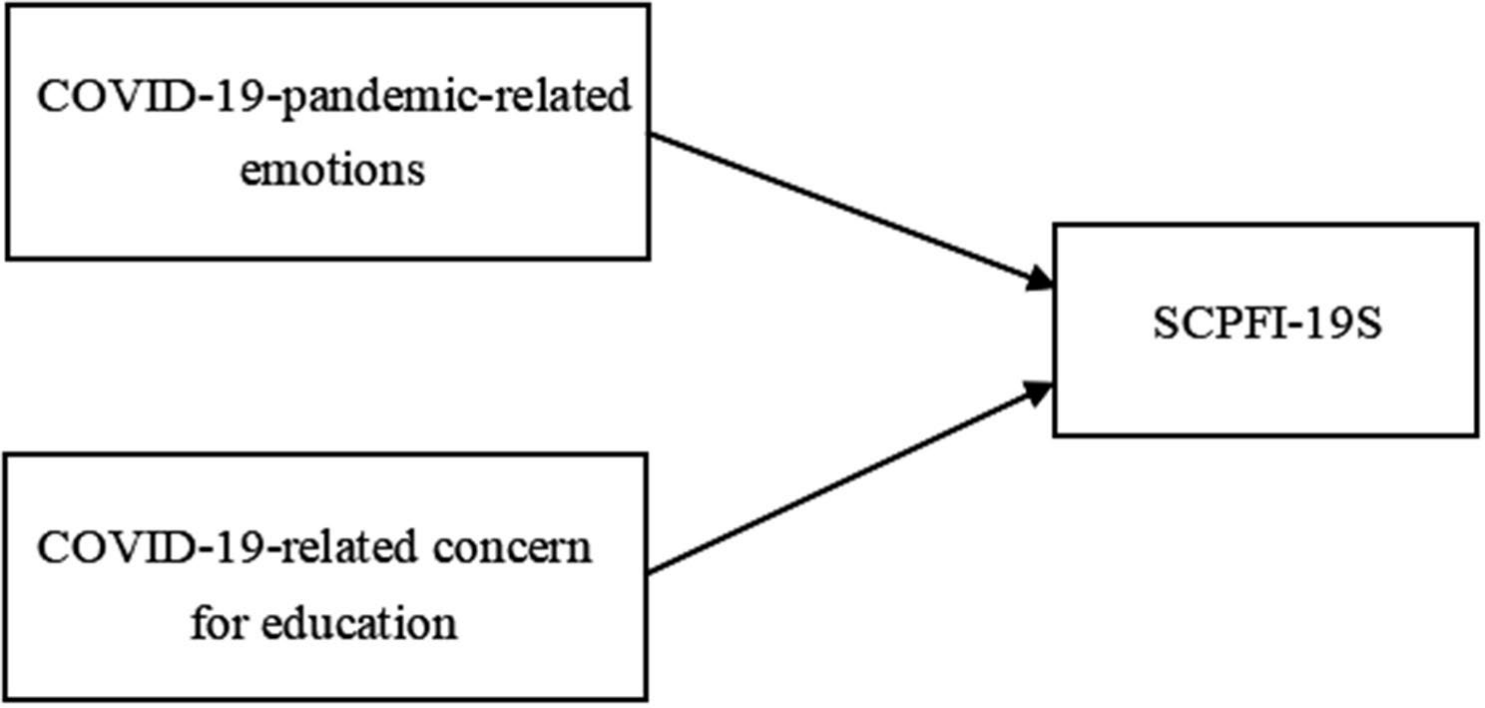
Hypothetical model

Based on published studies, existing literature and theories, we propose the following hypothesis: COVID-19-pandemic-related emotions and COVID-19-related concern for education in nursing students are significantly associated with SCPFI-19 S. (see Fig. 1). This study aims to provide a theoretical reference on how to effectively improve the academic development of nursing students.

## Materials and methods

### Participants

This study adopts a multi-stage sampling design, with a period of March–June 2023 to complete. Inclusion criteria: (1) enrolled as a full-time nursing student; (2) voluntary participation; and (3) ability to speak, read, and communicate effectively with the investigators. Exclusion Criteria: (1) refused to take part in this research; (2) non-nursing students; and (3) persons whose studies were interrupted or who were unable to complete their studies during the period of investigation. Based on the criteria proposed by Kendall, which require a minimum of 10 respondents per item, we calculated the sample size by multiplying the total number of items on the scale by 10 (N = [5 + 3 + 9] * 10 = 170) [21].

First, Jinzhou Medical University was randomly selected from six nursing colleges in Liaoning Province. Secondly, 80% of the classes were selected from each grade level (16 to 18 classes) in the university, including the high school undergraduate and vocational college undergraduate students. Thus, a total of 40 classes were selected from the school. Thirdly, 25–30 students were sampled from each class. A total of 1186 nursing students were selected. Trained interviewers conduct face-to-face interviews with nursing students. Prior to data collection, the 10 graduate students responsible for the interviews received uniform training on how to use standardized language and instructions. In the end, 1176 out of 1186 nursing students participated in the survey, of which 1126 effectively completed the questionnaire. The effective response rate was 94.94%, and the average time to complete the questionnaire was approximately 3.5 min. The study protocol met the standards and was approved by the Ethics Committee of Jinzhou Medical University, and informed consent was obtained from participants and legal guardians.

### Instrument

#### The COVID-19-pandemic-related emotions scale

The COVID-19-pandemic-related emotions scale was taken from Bellini et al.‘s recent study, which aimed to assess the psychological responses of nursing students to COVID-19 [22]. The scale consists of 5 items: fear, anxiety, worry, sadness, and anger. These items were scored on a Likert-type scale ranging from 1 (not at all) to 5 (extremely). The total score is obtained by summing the responses to each item, with scores ranging from 5 to 25. In this study, the Cronbach’s alpha of the scale was 0.932, indicating good internal consistency [23].

#### The COVID-19-related concern for education scale

Tone Nygaard Flølo et al. compiled the COVID-19-related concern for education scale in 2022 [24]. The scale consists of 9 items, which are: (1) trust in governmental handling of the COVID-19 situation; (2) trust in universities’ handling of the COVID-19 situation; (3) feeling lonely due to COVID-19 placement; (4) concerns about the quality of education; (5) concerns about high absenteeism during clinical training; (6) concerns about the completion of clinical training; (7) fewer learning situations during clinical training; (8) insufficient guidance during clinical training; (9) satisfaction with the educational curriculum. These items were scored on a 5-point Likert scale, where 1 = strongly disagree, 2 = disagree, 3 = neither agree nor disagree, 4 = agree, and 5 = strongly agree. The Cronbach’s alpha value for this scale in this study was 0.851, which has good internal consistency [25].

#### The impact of COVID-19 on study careers and professional futures scale

The impact of COVID-on the study careers and professional futures scale was developed by Tone Nygaard Flølo et al. [24]. The scale consists of 3 items, which were rated on a five-point Likert scale and ranged from “not at all” (1 point) to “extremely” (5 points). The total score of the scale ranged from 3 to 15. The higher the score, the more severe the effect. The Cronbach’s alpha of the scale was 0.940, which has good internal consistency [23].

### Statistical analysis

The factor structure of the scale was analysed in this study using exploratory factor analysis. Statistical analysis were performed using IBM SPSS 25.0; measurements were expressed as means and standard deviations; counts were expressed as frequencies and percentages; and the effect of COVID-19 on nursing students’ careers and professional futures was tested using t-tests or ANOVA. Pearson correlation analysis was used to describe the relationship between variables. A hierarchical multiple regression model was developed to test the incremental variance of each combination of independent factors, with SCPFI-19 S as the dependent variable. In this study, the first step added the basic characteristics of nursing students to the regression model, the second step added COVID-19-pandemic-related emotions to the regression model, and the third step added COVID-19-related concern for education to the regression model. The relative importance of the variables retained in the final multivariate regression model helped to explain the variance of the SCPFI-19 S, expressed as standardized beta values. Adjusted R^2^ values were used to assess model fit. P-values less than 0.05 (bilateral) were considered statistically significant.

## Results

### Exploratory factor analysis

The KMO test for the COVID-19-pandemic-related emotions scale was 0.849, and Bartlett sphericity test was significant (χ2 = 5116.307; P < 0.001). One factors supported by gravel maps accounted for 78.627% of the variance.

The KMO test for the COVID-19-related concern for education scale was 0.832, and Bartlett sphericity test was significant (χ2 = 6979.067; P < 0.001). Two factors supported by gravel maps accounted for 71.650% of the variance, respectively explaining 48.282% and 23.369%.

The KMO test for SCPFI-19 S was 0.748, and Bartlett sphericity test was significant (χ2 = 3147.507; P < 0.001). One factors supported by gravel maps accounted for 89.320% of the variance.

### Demographic information

Of the 1126 nursing students, 145 (12.9%) were male and 981 (87.1%) were female. The age range of nursing students was 15 30 years. The majority of participants (53.0%) lived in urban areas, 38.7% in suburban areas, and 8.3% in rural areas. As for the willingness to choose the nursing program, 56.8% of all participants chose the nursing program voluntarily, 24.2% were influenced by others to choose the nursing program, and 19.0% chose the nursing program for other reasons. When it comes to whether or not to pursue a nursing profession after graduation, 47.4% of the participants answered yes, 12.0% answered no, and 40.6% answered unsure.

### Description of SCPFI-19 S for nursing students

In this study, the Mean ± SD of SCPFI-19 S was (6.32 ± 2.95) points. When SCPFI-19 S was the dependent variable, the difference between the place of residence, willingness, and whether to engage in nursing after graduation was liberalized had statistically significant differences in all variables. See Table 1 for more details.

**Table 1:** Descriptive characteristics of SCPFI-19 S for nursing students (N = 1126)

Variables	Group	N(%)	Mean ± SD	*F/t*	*P*
Gender	Male	145(12.9)	3.020 ± 0.251	-0.312	0.755
	Female	981(87.1)	2.941 ± 0.094		
Age	15–20	195(17.3)	6.686 ± 2.973	2.163	0.115
	21–25	926(82.3)	6.241 ± 2.930		
	≥ 26	5(0.4)	7.400 ± 4.827		
Residence	Urban	577(53.0)	5.970 ± 2.959	10.462	0.001
	Suburb	436(38.7)	6.812 ± 2.928		
	Rural	93(8.3)	6.258 ± 2.686		
Grade	Freshman	511(45.4)	6.325 ± 2.984	2.429	0.089
	Sophomore	460(40.9)	6.467 ± 2.935		
	Junior	155(13.8)	5.865 ± 2.851		
Willingness	Voluntary Choice	640(56.8)	2.843 ± 0.112	5.641	0.004
	Influenced by others	272(24.2)	2.996 ± 0.182		
	Others	214(19.0)	3.137 ± 0.214		
Whether to engage in nursing after graduation	Yes	534(47.4)	6.084 ± 2.783	3.601	0.028
	No	135(12.0)	6.719 ± 3.444		
	Uncertain	457(40.6)	6.477 ± 2.965		

### Correlation of SCPFI-19 S variables with predictors for nursing students

In Pearson correlation analysis, COVID-19-pandemic-related emotions were positively correlated with SCPFI-19 S (*r* = 0.566, *P* < 0.01). The COVID-19-related concern for education was also positively correlated with SCPFI-19 S (*r* = 0.199, *P* < 0.01). See Table 2 for more details.

**Table 2:** Means, standard deviations, and correlations among the study variables

Variables	Mean ± SD	1	2	3
COVID-19-pandemic-related emotions	10.094 ± 4.359	1		
COVID-19-related concern for education	29.038 ± 6.487	0.185^**^	1	
SCPEI-19 S	6.320 ± 2.950	0.566^**^	0.199^**^	1

*Note*^∗∗^ represents *P* < 0.01

### Hierarchical multiple regression analysis

The results of the multiple linear regression analysis of the effect of COVID-19 on nursing students’ academic careers and professional futures are presented in Table 3. At each step, the independent variables contributed significantly to their variance. Using SCPFI-19 S as the dependent variable, the general demographic characteristics of nursing students were first examined, including gender, age, place of residence, grade, willingness, and whether to engage in nursing after graduation. These factors accounted for 1.4% of the variance. In the second step, after controlling for demographic characteristics, this study found that COVID-19 pandemic-related emotions were positively associated with SCPFI-19 S. COVID-19-pandemic-related emotions had a significant effect on nursing students’ study careers and professional futures (*F* = 78.012, adjusted *R*^*2*^ = 0.324, *R*^*2*^ change = 0.309). In the third step, “COVID-19-related concern for education " was added to the model, and it was significantly and positively associated with SCPFI-19 S explaining 33.3% of the variance. See Table 3 for more details.

**Table 3:** Hierarchical multiple regression prediction SCPFI-19 S scores

Variables	Step 1		Step 2		Step 3	
	*β*	*P*	*β*	*P*	*β*	*P*
Control variables						
Gender	0.013	0.652	-0.018	0.463	-0.023	0.355
Age	-0.041	0.175	-0.039	0.119	-0.038	0.122
Residence	0.098^***^	0.001	0.036	0.150	0.033	0.182
Grade	-0.027	0.368	-0.014	0.571	-0.004	0.862
Willingness	0.057	0.068	0.038	0.143	0.041	0.108
Whether to engage in nursing after graduation	0.048	0.126	0.039	0.128	0.042	0.101
COVID-19-pandemic-related emotions			0.561^***^	< 0.001	0.543^***^	< 0.001
COVID-19-related concern for education					0.101^***^	< 0.001
*F*	3.722^***^		78.012^***^		71.330^***^	
*R* ^*2*^	0.020		0.328		0.338	
Adjusted *R*^*2*^	0.014		0.324		0.333	
*R*^*2*^-change	0.020		0.309		0.010	

Note^***^p < 0.001

## Discussion

This study is the first to explore and determine the relationship between nursing students’ demographic characteristics, COVID-19-pandemic-related emotions, COVID-19-related concern for education, and their study careers and professional futures. This study is valuable for understanding how nursing students respond to public health event-related content and achieve holistic personal development.

Nursing students living in rural areas had the highest scores on the impact of COVID-19 on their study careers and professional futures. This may be due to the fact that, first, educational resources in rural areas are relatively limited, resulting in a large gap in their subject knowledge and skill base. In order to fill this gap, they often need to invest more time and effort; second, rural areas may be relatively underdeveloped in terms of internet and technological facilities, which may make distance learning and online education more challenging. If nursing students are not able to fully participate in distance learning, their educational experience and the quality of their learning may be affected [26].

Students who chose the nursing program for other reasons scored highest on the impact of COVID-19 on their study careers and professional futures. During the epidemic, care workers faced greater occupational risk due to the high contagiousness of COVID-19, which may have caused them to doubt their choice of nursing program or re-evaluate career options. In addition, the epidemic may have led to uncertainty in the job market, including the nursing profession. They may be more concerned about future employment stability and opportunities, which may add to their academic and psychological burdens, and they may face greater challenges during their educational years [27].

Students who did not pursue nursing after graduation scored significantly higher on the impact of COVID-19 on their study careers and professional futures than those who were engaged in or unsure of their nursing careers after graduation. This may be due to the fact that the fluctuations in the job market and changes in the industry during the epidemic made these students feel more troubled and unsure of their career direction, which in turn affected academic focus and motivation [28, 29].

Since the beginning of the COVID-19 pandemic, several investigations have been carried out to assess the impact of this emergency on healthcare professionals, with particular attention paid to the psychological impact [29, 30]. Several recent studies have reported data on the impact of the SARS-CoV-2 emergency on the academic education of future nurses. It has been shown that the epidemic led to an increase in students’ negative feelings towards the nursing profession, higher levels of anxiety, and a reluctance to pursue a nursing career in the future [31, 32]. The results of this study indicated a significant positive correlation between nursing students’ emotions related to their study careers and professional futures during the COVID-19 pandemic. Emotions have a profound impact on learning [33]. Jian Yang et al. found that uncertainty about the COVID-19 pandemic and concerns about their own health may increase anxiety and stress among nursing students [34]. This emotional state may affect their concentration, learning efficiency, and even their performance in the field of education. Mai Sakai’s study confirmed that social distance and segregation measures may cause nursing students to feel lonely and isolated by their peers and teachers [35]. This loneliness may exacerbate their emotional distress and affect social support in the academic and educational process. Magdalena Dziurka’s research indicated that nursing students may feel worried and sad if they are unable to gain sufficient clinical practice experience or face educational challenges due to the impact of the epidemic. Such emotions may reduce their motivation and commitment to their educational endeavors [36]. These findings supported the results of this study. Despite the negative mental health impact of the epidemic on nursing; on the other hand, individuals have an intrinsic motivation to grow positively by seeking to implement effective ways to cope. It has been shown in the past that the application of positive psychology can help people maintain a stable mental state, even when facing difficulties. Secondly, every student should develop the ability to approach problems from multiple perspectives, find solutions, and employ positive thinking to cope with challenges in their academic and personal lives, contributing to their overall development in education [23]. Therefore, it is critical to explore in depth the relationship between emotions associated with the COVID-19 pandemic and nursing students’ study careers and professional futures. By gaining a deeper understanding and examining this impact, we can implement practical interventions to help students better cope with their emotions, thereby contributing to their overall development in education.

The results of the study showed a positive correlation between nursing students’ concern for education and their impact on learning careers and career futures during the COVID-19 pandemic. That is, the higher the education concern score, the higher the impact on study careers and career futures score. Li Z-S et al. found that even under normal circumstances, nursing students may be exposed to higher levels of stress than students in other health professions and therefore may be particularly vulnerable to educational constraints resulting from measures taken to limit the spread of COVID-19 [37, 38]. Clinical training is an essential component of all nursing education. Due to the limitations of the epidemic, nursing students may face fewer learning situations and insufficient instruction in possible clinical training [24, 39]. Some schools may use distance learning, which may not be as effective for nursing students as field participation in clinical practice [40]. Distance learning may not provide the experience of direct interaction with patients, limiting the development of practice skills. However, An H et al. showed that a lack of clinical practice experience may prevent nursing students from providing sufficient examples and case studies in their teaching, leading to relatively theoretical content and a lack of practical support, which ultimately affects the students’ integrated learning experience [41, 42]. However, some research suggests that moderate educational worry can motivate students to study further and harder, leading them to be more focused and engaged in their academics. Excessive worry may result in students’ academic development being hindered [13]. Therefore, a balance between educational concerns and academic development is critical. Schools can help students achieve good academic outcomes through positive communication, personalized support, and attention to students’ mental health.

However, there are some limitations to our study. First, due to the cross-sectional design and self-report measures, we were unable to determine the causal relationship between COVID-19-pandemic-related emotions, COVID-19-related concern for education, and study careers and professional futures. The relationship between these variables should be further explored using a longitudinal approach in future studies. Second, our study sample represented nursing students from a specific city, which may not be fully representative of the reality of the entire nursing student population. Future studies should consider expanding the sample size by including more regions and various types of nursing educational institutions to enhance the applicability and generalizability of the findings.

Despite limitations, our findings provided an overview of the situation of undergraduate nursing students after the COVID-19 blockade and suggested possible intervention strategies to protect their well-being. First, and most important, is to maintain a stable educational framework, including minimizing changes in teaching programs and publishing letters about changes as soon as possible [43]. Second, university-specific interventions (e.g., psychoeducation, emotional self-regulation, and positive mental health promotion) to address students’ biopsychosocial needs [44]. Finally, methods to increase self-esteem, sense of self-control, and self-efficacy may enhance students’ control over their clinical practice [45]. Nursing students are the future health care providers, and their education and healthy growth are important for the development of the health care industry [46].

Theoretically, this study contributes to a deeper understanding of the mechanisms by which the emotional and educational concerns of nursing students in the epidemic affect their study careers and professional futures. Practically, this study provided new perspectives on how to promote nursing students’ academic careers and professional futures. At the same time, this study can also guide us to learn lessons from this COVID-19 epidemic, pay attention to solving the problems of nursing students in public health emergencies, better meet the needs of nursing personnel training in the COVID-19 epidemic, and further improve the system of nursing personnel training in vocational schools.

### Application of research findings to education and clinical nursing practice

COVID-19 epidemics, as acute, pervasive, and persistent stressors, have a significant impact on both society and individuals. This study was conducted to correlate the emotions, educational concerns, and impact on the academic career and professional future of nursing students during the epidemic. It was also to understand the factors affecting nursing students’ academic careers and professional futures. These findings not only provide substantial suggestions for students to better address their emotional concerns but also provide guidance for improving nursing education, thus providing a theoretical reference for nursing educators to improve the academic development of nursing students in higher education.

## Conclusion

Overall, both COVID-19-pandemic-related emotions and COVID-19-related concern for education were significantly associated with both study careers and professional futures. In the future, education systems will need to be flexible and adaptive to change, focusing on the holistic development and mental health of students to ensure that they have access to effective education and support despite epidemics and other challenges. At the same time, educational institutions and policymakers should consider the needs of students in changes to teaching and assessment models to ensure that changes do not negatively affect students’ academic progress, especially in practice-oriented professions such as nursing.

## Data Availability

The datasets used and analyzed during the current study are available from the corresponding author upon reasonable request.

## Abbreviations

SD: Standard Deviation
SCPFI-19 S: The impact of COVID-19 on the study careers and professional futures scale
β: Standardized
